# Dietary status with demographic and anthropometric variables and some health affecting risk factors in people of Southeastern Iran: A population-based study (KERCADRS)

**DOI:** 10.22088/cjim.12.4.551

**Published:** 2021

**Authors:** Hamid Najafipour, Farzaneh Abdollahi, Mojgan Khatibi, Raheleh Amirzadeh

**Affiliations:** 1 Cardiovascular Research Center, Institute of Basic and Clinical Physiology Sciences and Department of Physiology, Kerman University of Medical Sciences, Kerman, Iran; 2 Endocrinology and Metabolism Research Center, Institute of Basic and Clinical Physiology Sciences, Kerman University of Medical Sciences, Kerman, Iran; 3 Physiology Research Center, Institute of Neuropharmacology, and Department of Nutrition, Kerman University of Medical Sciences, Kerman, Iran; 4 Research Center for Social Determinants of Health, Institute for Future Studies in Health, Kerman University of Medical Sciences, Kerman, Iran

**Keywords:** Dietary patterns, Prudent diet, Risk factors, Health outcomes, Southeastern Iran

## Abstract

**Background:**

Dietary pattern is influenced by lifestyle, genetic, cultural, and socioeconomic factors. We investigated the status of prudent (PDP) and imprudent (IDP) dietary patterns and their relationship with demographic and anthropometric variables and health conditions in an urban population of Southeastern Iran.

**Methods:**

The study was conducted on 9997 people aged 15-80 years randomly selected using one-stage cluster sampling. Demographic and anthropometric measurements were recorded through face-to-face interview, and their nutritional status was assessed by the food frequency questionnaire. PDP and IDP were identified based on scoring to the type and daily/weekly frequency of foods consumed and their production methods.

**Results:**

Fifty nine percent of the participants were females. PDP participants reported daily intake of whole grains (99.5%), fruits (66.5%), and unsaturated oil (88.6%). Daily intake of sweets and high fat dairy products were 55.7% and 46%, respectively. Women (64.8% vs 35.2 %) and people with diabetes (p<0.001), hypertension (p<0.01), and higher BMI (p<0.02) had healthier dietary status. People with lower education, cigarette smokers and opium users had a higher rate of IDP (all p<0.001). The dietary pattern improved with aging (AOR of IDP decreased from 1 in 15-24 years to 0.20 for 65-75 years) (p<0.001).

**Conclusion:**

Younger people, men and those with lower education had unhealthier dietary pattern. It seems that dietary behavior is more related to the individuals' tendencies and taste preferences. Modification of nutritional behaviors of the population and leading young people, men, and those with lower education to improve their dietary pattern is recommended.

Diet and nutrition play an important role in health and in the prevention of diseases, and low-quality diet is one of the leading causes of mortality and morbidity worldwide (1). For many years, studies conducted on the human nutrition and its effect on the human health have emphasized the effect of a type of food, but nowadays, nutritionists believe that since human uses combinations of foods, therefore, identification of dietary patterns as factors affecting the physical and mental health, is of great importance (2). Dietary pattern also reflects the individual’s tendencies and taste preferences. The dietary patterns are affected by various factors including lifestyle, genetics, culture, and socioeconomic conditions (3, 4). Iran, like other countries in the world, has experienced undesirable changes in the dietary intake as a result of industrialization and nutrition transition. 

Therefore, modification of dietary patterns may lead to positive health consequences (5). In many studies, dietary patterns are usually divided into two categories (6):

1. Prudent (healthy) dietary pattern (PDP) includes preferential intake of low-fat dairy products, vegetables, fish, whole grains, poultry, a high intakes of fruits and vegetables, and low consumption of processed and fermented meat. 

2. Imprudent (unhealthy) dietary pattern (IDP) includes preferential intake of red and processed meat, fried potatoes, refined grains, snacks, high-fat dairy products, sweets and fast foods and low intakes of fruits and vegetables. 

The Australian Dietary Guidelines support the following dietary patterns to reduce the risk of cardiovascular disease (CVD) and type 2 diabetes: 1) vegetarian-based diet including intake of fruits, vegetables, legumes and whole grains, and 2) animal-based diet including intake of white meat, fish, poultry, low-fat dairy products, restricting the intake of saturated oils, choosing low-sodium foods, moderate intake of sugar, and restricting alcohol consumption (7). However, Australians intake is usually small in the amounts of fruits and vegetables, but eat large amounts of processed meat and processed foods.

Some studies have assessed the relationship between dietary patterns and the incidence of diseases or their effects in the human health. Overall global diets are largely shifting toward processed foods high in refined carbohydrates and added sugars and away from legumes, coarse grains, and other vegetables (8). Low- and middle-income countries face a rapid change in the nutrition transition toward increase in the prevalence of non-communicable diseases such as overweight/obesity, diabetes and hypertension. Popkin et al. showed overall shifts in diet, physical activity and body composition in developing countries towards unhealthier conditions (9). 

In a study on dietary patterns conducted in Khorramabad, Iran by Falahi et al., (2013), a nutrition transition from traditional to western diet and snacks was reported (10). Today, the intake of snacks is relatively high among teenagers and adolescents in western countries (11) and dietary pattern of many Iranians needs to be modified (12).

To the best of our knowledge, limited information existed on current dietary patterns of the population in southeastern Iran and its relationship with demographic, anthropometric and health related variables especially less traditional risk factors/risk behaviors such as smoking, opium use, low physical activity, anxiety and depression. Therefore, the aim of this large population-based study was to investigate the dietary status of people aged 15-80 years in a representative urban population in Southeast Iran and its association with demographic, anthropometric and some health-related variables. The results can help health authorities to adopt the strategies required for health planning and modify the inappropriate dietary patterns if present. 

## Methods

This is a sub-analysis of the data from a cross-sectional study on the prevalence of coronary artery disease (CAD) risk factors on 9997 people aged 15-80 years between 2014 and 2018, called Kerman Coronary Artery Diseases Risk Factors Study (KERCADRS, phase 2), in Kerman the largest city in Southeastern Iran. Nutrition status was assessed as one of the CAD risk factors in KERCADRS. Kerman is the capital of Kerman Province about 1000km far from the capital city, Tehran. Its population is about 750,000 inhabitants (National census, 2016). People are mostly busy with white-collar works at governmental sections, agriculture and marketing. The lifestyle patterns are typically aggregated in families. The study was conducted according to the guidelines in the Declaration of Helsinki and all procedures were approved by the Ethics Committee of Kerman University of Medical Sciences (Permission code: IR.KMU.REC.1392.405). Subjects were selected using one-stage cluster sampling method and written informed consent was obtained from all participants. 

The inclusion criteria was age between 15-80 years and residency in Kerman for at least one year prior to interview. More details about study protocols and sampling methods which are similar to phase 1 are found in the previous publication about methodology of that phase (13). Concisely, using the city zip code list in the post office, 420 zip codes were randomly selected. From each zip code 24 subjects (12 males and 12 females in the range of 15-80 years old) were invited to the study by attending their addresses. The subjects were referred to a site located in the city center and their demographic characteristics (including age, sex, level of education, smoking, and opium use) were recorded through face to face interview. Anthropometric measurements were, height and waist circumference (measured by a tape stadiometer, with a minimum measurement of 0.1 cm in a standing position without shoes, and weight (light clothing without shoes measured by a standard weighing scale (Seca, Germany) with an accuracy of 100 g. The BMI was divided into three classes: normal up to 24.9 kg/m^2^), overweight (25-29.9 kg/m^2^), and obese (≥30 kg/m^2^) (14). Any individual who was previously diagnosed with DM and/or was taking insulin or non-insulin drugs and/or had fasting plasma glucose ≥126 mg/dL at the time of recruitment was considered diabetic (13). HTN was defined as a systolic blood pressure ≥140 mm Hg and/or a diastolic blood pressure ≥90 mm Hg and/or taking any antihypertensive drug (13). Dyslipidemia was defined as total cholesterol ≥200 mg/dL and/or LDL>130 mg/kg, and/or HDL<30 mg/kg in men or HDL<45 mg/kg in women. Hypertriglyceridemia was defined as TGs≥200 mg/dL (13). Physical activity was measured using the Global Physical Activity Questionnaire, and metabolic equivalent of task (MET) was used to express the intensity of physical activity, as low (less than four times of the energy consumed at rest), moderate (between 4 -8 times relative to the rest) and intense (more than 8 times relative to the rest) (15). 

To assess the nutritional status of the participants, the food frequency questionnaire (FFQ) was used (16). Validity and reliability of the questionnaire had been determined in the previous studies (17). To determine dietary pattern, two PDP and IDP dietary patterns were identified (18). The authors also consulted with two nutritionists to classify the dietary patterns into two groups of PDP (healthier) and IDP (unhealthier). PDP includes consuming nine food groups (fruits, vegetables, whole grains, nuts and seeds, low-fat dairy products, legumes, unsaturated oil, white meat and boiled foods) and IDP includes 11 food groups (high-fat dairy products, refined grains, fried foods, butter, cream, fats, salt, beverages, sweets, red meat, and fast foods).

Then, the consumption frequency was scored into: daily consumption = 2, weekly consumption (once a week) = 1, monthly consumption (once a month) and less = 0 (total max score of 18 for PDP and total max score of 22 for IDP dietary patterns). Finally, the total score for each participant was calculated and he/she was assigned to PDP or IDP based on his/her total score. 

As a participant with high score od PDP may simultaneously have a low score of IDP and vice versa, to eliminate the overlap between the scores of PDP and IDP, from 22 scores of IDP the scores less than 7 were included to PDP diet group and scores between 8 and 22 were considered as IDP diet. Correspondingly, from 18 scores of PDP the scores less than 6 were included to IDP group and scores between 7 and 18 were considered as PDP diet. Therefore, PDP included participants with upper two-third scores of healthy food consumption and the lower third scores of unhealthier food consumption, and IDP included participants with upper two-third scores of unhealthy food consumption and lower third scores of healthier food consumption (18).

Data were analyzed using descriptive statistics and chi-square test, and the relationship between dietary patterns and demographic variables was determined using logistic regression analysis (SPSS Version 21). Statistically, the significant level was considered at P≤0.05.

## Results

In the present study, among the 10,000 subjects that participated, 59% were females. The mean±SD of BMI was 25.8±4.6 kg/m^2^ in males and 27.9±5.1 kg/m^2^ in females (p<0.01). Table 1 shows the frequency of food groups’ intake in the participants with PDP and IDP. Regarding the use of PDP, participants reported daily intake of whole grains (99.5%), unsaturated oil (88.6%) and fruits (66.5%). But only 25% of the PDP participants reported daily intake of low-fat dairy products and 19.4% reported daily intake of vegetables. Regarding the use of IDP, participants reported daily intake of sweets (83.6%), fried foods (70.8%), high-fat dairy products (66%), and saturated oil (44.9%). At least 5.3% of the participants added salt at the table daily (table 1). The use of red meat, saturated oil and fried foods was almost three times in IDP group compared to PDP group, as it was 10 times for soft drinks.

Table 2 shows the association between demographic variables (gender, age, education, and BMI) and dietary pattern via chi-square test. Overall, 36.4% of the participants in the study followed IDP. 64.8% of women compared to 35.2% of men had PDP (p<0.001). There was a significant difference in terms of education, BMI, and age between the PDP and IDP (p<0.001). Higher educated people are more in PDP and illiterates are more among IDPs. With increasing age, people moved toward healthier patterns and reduced the unhealthy patterns (table 2). Obese and centrally obese subjects adhere more with PDP compared to IDP (23% vs 20.5% and 32.3% vs 28.6% respectively, p<0.001).

Normal BMI <25, Overweight: 25 ≤ BMI < 30, and Obese: BMI ≥ 30. Central obesity (inappropriate WC) was defined as WC >88 cm for women and WC >102 cm for men; KERCADRS: Kerman coronary artery diseases risk factors study. Table 3 shows the odds ratios of IDP with demographic variables using logistic regression. The adjusted odds ratio (AOR) of the IDP in people with higher education was 0.59 (CI: 0.48-0.73). AOR of IDP decreased with increasing age and it was 0.58 for diabetics, 0.79 for hypercholesterolemic and 0.85 for hypertensive subjects. AOR of IDP was significantly higher in cigarette smokers and opium addicts (1.3 and 1.4 respectively). Younger ages and male gender were risk factors for having IDP.

**Table 1:** The Frequency distribution (%) of different foods consumption in people with Prudent (healthier) dietary pattern (PDP), and imprudent (unhealthier) dietary pattern (IDP), Community-Based Study (KERCADR –2nd Phase- n=9997), Kerman, Iran, 2014-2018

**Subgroups**	**%People with PDP** **(n= 6360) **	**%People with IDP (n=3635)**	**P. value**
**Low-fat dairy products**			
Daily use	25.1	12.8	<0.0001
Weekly use	22.7	19.0	
Monthly /Never use	52.2	68.1	
**White meat**			
Daily use	0.5	0.8	0.20
Weekly use	92.0	91.6	
Monthly /Never use	7.5	7.6	
**Whole grains**			
Daily use	99.5	98.8	<0. 01
Weekly use	0.2	0.2	
Monthly /Never use	0.3	0.9	
**Nuts/seeds**			
Daily use	11.6	8.5	<0.0001
Weekly use	53.0	53.3	
Monthly /Never use	35.4	38.2	
**Fruits**			
Daily use	66.4	60.0	<0.001
Weekly use	30.9	34.6	
Monthly /Never use	2.7	5.4	
**Vegetables**			
Daily use	19.4	14.9	<0.001
Weekly use	55.8	56.3	
Monthly /Never use	24.8	28.9	
**Legumes**			
Daily use	2.7	4.6	<0.001
Weekly use	90.5	89.4	
Monthly /Never use	6.9	5.9	
**Boiled foods**			
Daily use	49.0	10.6	<0.0001
Weekly use	27.4	23.9	
Monthly /Never use	23.6	65.5	
**Unsaturated oil**			
Daily use	88.6	64.0	<0.0001
Weekly use	5.0	10.2	
Monthly /Never use	6.4	25.8	
**High-fat dairy products**			
Daily use	45.9	66.0	<0.0001
Weekly use	28.8	25.8	
Monthly /Never use	25.3	8.1	
**Red meat**			
Daily use	1.5	4.8	<0.0001
Weekly use	82.5	85.7	
Monthly /Never use	16.0	9.5	
**Refined grains**			
Daily use	0.3	2.2	<0.0001
Weekly use	8.4	29.6	
Monthly /Never use	91.3	68.2	
**Saturated oil**			
Daily use	11.5	44.9	<0.0001
Weekly use	6.8	9.7	
Monthly /Never use	81.7	45.9	
**Butter/cream**			
Daily	0.8	4.4	<0.0001
Weekly	19.6	49.5	
Monthly /Never	79.5	46.1	
**Sweets**			
Daily use	55.7	83.6	<0.0001
Weekly use	18.1	12.2	
Monthly /Never use	26.2	4.2	
**Soft drinks**			
Daily use	1.2	12.3	<0.0001
Weekly use	22.4	55.0	
Monthly /Never use	76.5	32.6	
**Fast food**			
Daily use	0.1	1.3	<0.0001
Weekly use	7.0	32.8	
Monthly /Never use	92.9	65.9	
**Salt on table**			
Daily use	1.6	3.7	<0.0001
Weekly use	53.6	71.0	
Monthly /Never use	44.8	25.3	
**Fried foods**			
Daily use	25.2	70.8	<0.0001
Weekly use	28.7	20.7	
Monthly /Never use	46.1	8.5	
**Alcohol**			
Daily use	0.0	0.3	<0.001
Weekly use	0.4	1.7	
Monthly /Never use	99.6	98.0	

**Table 2:** The standardized prevalence of subgroups with prudent (healthier) dietary pattern (PDP), and imprudent (unhealthier) dietary pattern (IDP), Community-Based Study (KERCADR –2nd Phase- n=9997), Kerman, Iran, 2014-2018

**Subgroups**	**PDP** **diet** **% (95% CI) (n=6360)**	**IDP diet** **% (95% CI) (n=3635)**	**P. value**
**Overall**	63.6 (62.4 -64.5)	36.4 (35.4-37.3)	
**Sex**			
Men	35.2 (33.6-36.8)	47.4 (45.7-49.0)	<0.0001
Women	64.8 (63.1-66.3)	52.6 (50.9-54.2)	
**Age group (year)**			
15-24	5.4 (4.9-6.4)	13.9 (12.8-15.0)	<0.0001
25-34	12.8 (12.0-13.7)	24.3 (22.9-25.8)	
35-44	18.6 (17.7-19.6)	23.0 (21.7-24.4)	
45-54	22.1 (21.1-23.1)	17.3 (16.1-18.6)	
55-64	24.6 (23.6-25.7)	13.2 (12.2-14.4)	
65-80	16.4 (5.5-17.3)	7.9 (7.0-8.8)	
**Education**			
Illiterate	4.3 (3.9-4.7)	4.6 (4.1-5.1)	<0.0001
Primary to high school	69.1 (67.6-70.6)	75.4 (74.0-76.9)	
Above high school	26.4 (24.9-27.9)	19.8 (18.5-21.1)	
**Body Mass Index**			
<25	41.0 (39.2-42.3)	44.5 (42.9-46.1)	<0.0001
25-29.9	36.0 (34.5-37.5)	35.0 (33.2-36.3)	
≥30	23.0 (21.8-24.3)	20.5 (19.2-21.8)	
**Waist circumference**			
Normal	67.7 (66.4-69.1)	71.4 (70.0-72.8)	<0.0001
Inappropriate	32.3 (30.8-33.5)	28.6 (27.1-29.9)	
**Current cigarette smoker**			
No	94.7 (94.1-95.3)	89.3 (88.3-90.2)	<0.0001
Yes	5.3 (4.6-5.8)	10.7 (9.7-11.6)	
**Opium use**			
No	91.4 (90.6-92.0)	83.2 (82.1-84.3)	<0.0001
Occasional user	6.1 (5.6-6.7)	12.1 (11.1-13.1)	
Depended user	2.5 (2.0-2.9)	4.6 (3.9-5.2)	
**Depression**			
No	85.0 (83.7-86.0)	83.6 (82.4-84.8)	0.169
Yes	15.0 (13.9-16.2)	16.3 (15.1-17.5)	
**Anxiety**			
No	60.0 (58.3 -61.5)	57.1 (55.4-58.7)	0.021
Yes	40.0 (38.4-41.6)	42.8 (41.2-44.5)	
**Diabetes**			
Normal	78.1 (77.0-79.2)	81.1 (79.9-82.3)	<0.0001
Pre-Diabetic	11.8 (10.9-12.8)	13.0 (11.9-14.1)	
Diabetic	9.9 (9.3-10.6)	5.7 (5.0-6.4)	
**Hypertension**			
Normal	87.0 (86.4-87.6)	89.6 (88.6-90.3)	<0.0001
Hypertensive	12.9 (12.3-13.5)	10.4 (9.6-11.3)	
**Hypercholesterolemia**			
Normal	84.6 (83.7-85.4)	89.0 (88-89.8)	<0.0001
Hypercholesterolemia	15.4 (14.5-16.2)	11.0 (10.1 -11.9)	
**Hypertriglyceridemia**			
Normal	69.0 (67.7-70.3)	71.5 (70.1-72.9)	<0.0001
Hypertriglyceridemia	31.0 (29.6-32.2)	28.4 (27.0-29.8)	
**Physical activity**			
Low	46.5 (44.7-48.0)	47.6 (45.8-49.1)	<0.0001
Moderate	36.5 (34.8-37.1)	37.0 (36.3-39.6)	
High	17.0 (15.7-18.2)	15.4 (14.3-16.8)	

**Table 3:** Crude and Adjusted odds ratio (AOR) for different associated factors of imprudent (unhealthier) dietary pattern (IDP), Community-Based Study (KERCADR –2nd Phase n= 9997), Kerman, Iran, 2014-2018

**Subgroups**	**IDP diet**		**P. value** **Adjusted**
	**Crude OR**	**Adjusted OR**	
**Sex**			
Men	1	1	<0.0001
Women	0.62 (0.57-0.68)	0.65 (0.59-0.73)	
**Age group (year)**			
15-24	1	1	
25-34	0.74 (0.62-0.87)	0.80 (0.67-0.95)	0.012
35-44	0.48 (0.41-0.57)	0.50 (0.42-0.59)	<0.0001
45-54	0.30 (0.26-0.36)	0.32 (0.27-0.39)	<0.0001
55-64	0.21 (0.17-0.25)	0.23 (0.19-0.28)	<0.0001
65-75	0.18 (0.15-0.22)	0.20 (0.16-0.25)	<0.0001
**Education**			
Illiterate	1	1	
Primary to high school	1.7 (1.5-2.0)	0.87 (0.73-1.04)	0.13
Above high school	1.4 (1.2-1.7)	0.59 (0.48-0.73)	<0.0001
**Body Mass Index**			
Normal	1	1	
overweight	0.66 (0.60 -0.73)	0.88 (0.79-0.98)	0.029
obese	0.57 (0.51-0.63)	0.83 (0.71-0.97)	0.020
**Waist circumference**			
Normal	1	1	
Obese	0.63 (0.58-0.69)	1.1 (1.0-1.2)	0.036
**Current cigarette smoker**			
No	1	1	
Yes	1.7 (1.4-1.9)	1.3 (1.1-1.5)	<0.0001
**Opium addiction**			
No	1	1	
Occasional user	1.4 (1.2-1.6)	1.6 (1.3-1.8)	<0.0001
Depended user	1.8 (1.5-2.3)	1.4 (1.1-1.8)	<0.0001
**Depression**			
No	1	1	
Yes	1.08 (0.96-1.2)	1.1 (0.97-1.2)	0.120
**Anxiety**			
No	1	1	
Yes	1.1 (1.0-1.2)	1.1 (1.0-1.2)	0.004
**Diabetes**			
No	1	1	
Pre-Diabetic	0.8 (0.71-0.89)	1.0 (0.9-1.1)	0.613
Diabetic	0.3 (0.27-0.35)	0.58 (0.4-0.6)	<0.0001
**Hypertension**			
No	1	1	
Yes	0.41 (0.36-0.45)	0.85 (0.74-0.97)	0.016
**Hypercholesterolemia**			
No	1	1	
yes	0.41 (0.36-0.45)	0.76 (0.66-0.88)	<0.0001
**Hypertriglyceridemia**			
No	1	1	
Yes	1.6 (1.5-1.8)	0.97 (0.87-1.0)	0.57
**Physical activity**			
High	1	1	
Moderate	0.77 (0.68-0.87)	1.0 (0.89-1.1)	0.716
Low	0.82(0.73-0.92)	1.0 (0.92-1.1)	0.449

## Discussion

In this study, two dietary patterns of prudent dietary pattern (PDP) (fruits, vegetables, whole grains, nuts/seeds, low-fat dairy products, unsaturated oil and legumes) and imprudent dietary pattern (IDP) (high-fat dairy products, refined grains, fried foods, butter/cream, saturated fats, salt, beverages, sweets, red meat, and fast foods) were evaluated in about 10,000 of the adults living in a representative urban population in Southeast Iran. According to the results, overall, about 36% of the population follow IDP. There was a direct association between education, age, BMI, diabetes, hypertension and hypercholesterolemia with the adherence to PDP (table 2). Also there was a reverse relationship between anxiety, smoking and opium use with the adherence to PDP. Women follow more PDP than men.

The unexpected finding in the present study that the overweight, obese, and hypertensive people had more adherence to PDP may be with increasing BMI or becoming hypertensive, people were encouraged/recommended to use PDP. As the results of table 3 show, the odds of having IDP for diabetics, hypercholesterolemic and hypertensive participants were 0.58, 0.76 and 0.85 respectively. In agreement with our findings, in a study by Crovetto et al. (2018) on college students, underweight male students reported higher intake of unhealthy diet (19) and McNaughton et al. (2008) reported no significant relationship between BMI and dietary patterns (20). A study in 2013 in Pennsylvania on the association of healthy dietary pattern with obesity and mortality showed that in elderly people except for hypertension, no significant associations were found for CVD, diabetes mellitus, metabolic syndrome and mortality with dietary patterns (21). 

The other effective factor may be the prevalence of cigarette smoking and opium use in IDP group is double compared to DDP group (table 2). We have shown that the odds of overweight/obesity decreases significantly with smoking (AOR: 0.4) and opium use (AOR: 0.50) (14). In agreement to our findings, in a large cohort population study including 50,000 participants, with long follow-up time, in Iran, the people in the highest level of DASH score (a healthy dietary pattern) had higher BMI and a history of diabetes or hypertension (22). Furthermore, they found no association between the DASH diet pattern and risk of CVD mortality. It seems that the relationship between dietary pattern and CVD risk factors and outcomes is more complex and probably more related to the cultural and lifestyle diversities of the studied populations. The third possibility is that the prevalence of diabetes and obesity is higher in female gender (14), meanwhile females are the majority of participants in the present study and adhere more to PDP. This may partly shift the results towards the association of diabetes and PDP.

Another determinant factor associated with the use of PDP was the age of participants. According to the results, the pattern of healthier food intake improved (and unhealthier food intake reduced) with aging (tables 2 and 3). This means that older generations use healthier dietary patterns, unfortunately, younger individuals have more tendency towards IDPs. The reason for more adherence of younger generation to IDP may be the weakening of family relationships and they spend more time out of the house. Also, nowadays mostly both males and females work outside the home and there is less time remained to spend in making home foods. Unlike to the finding of the present study, a study by Assmann (2015) showed that there was no relationship between healthy aging and western dietary patterns. Besides, in people with higher energy intake who use PDPs, there was no relationship between PDP and healthy aging, while in those with low energy intake, there was a significant relationship between PDP and healthy aging (23). Therefore, in addition to the dietary pattern, it seems that the level of energy intake is another important determinant of health. Unfortunately, low physical activity is rising quicker in younger generation of the study population (15), and this in concert with inappropriate food intake will dispose them to increase the prevalence of CVD risk factors soon.

 It was expected that more educated people adhere more to PDP. In agreement with our results, several studies reported educational status as one of the factors encouraging people to use PDPs. Deshmukh-Taskar et al. (2009) and Eckel et al. (2005) have shown the positive effect of educational status on the use of PDP (24,25). A study by Siu (2018) in Hong Kong showed that there was a direct relationship between inappropriate dietary patterns in children and low parental educational levels (26). Conversely, we found one study performed by Dewayani (2018) in Spain, in which there was no relationship between educational status and the level of nutritional awareness among parents of primary school children and the intake of snacks by the children (27). 

Dietary pattern includes the amount and frequency of food groups’ intake. There are studies that along with the type of dietary pattern evaluated the food groups’ intake (28, 29). In this study, the adherence to food groups in each dietary pattern was heterogeneous, so daily intake of whole grains (99.5%), fruits (66.5%), and unsaturated oils (88.6%) by the majority of the PDP group indicates the attention of this population to healthier diet, while daily intake of sweets (56%) and high fat dairy products (46%), which are subgroups of IDP, was also relatively high in this population. It seems that the selection of food groups is more related to the tendencies and taste preferences of the study population, rather than merely being an indication of their awareness and behavior. This means that the tendency to use whole grains, fruits or vegetables, as subgroups of PDP, along with the tendency to use high fat dairy products and sweets, as subgroups of IDP, are more likely attributed to the habits and preferences of the study population, and cannot be just related to lack of literacy or inadequate knowledge. 

On the other hand, the mother who is the main decision maker in using food groups and cooking methods in Iran, should take into consideration the taste preferences of all family members in her decision-making. According to table 1, the prevalence of daily intake of white meat is less than red meat, while urban population are mostly aware that white meat is a healthier food. Probably, greater variety of red meat-based foods and traditional tastes of people, encourage her to use this product more frequently. On the other hand, the effect of official education and cyberspace information about the community health cannot be ignored, as we observed that educated people had a healthier diet. Also due to low socioeconomic status of the majority of the population in this area of Iran, many mothers in selecting foods consider “what they can afford”, not necessarily “what they wish”. It has been shown that economic access to foods is an important factor for adherence to a healthy diet (30).

We acknowledge the limitation of our study as a cross-sectional survey in exploring causality between dietary patterns and risk factors. Besides, this study benefited from a large sample size, random sampling, and wide age range of the studied population. For further studies, we recommend monitoring the dietary patterns in a context of a longitudinal prospective study to explore the exact effect of dietary pattern on the prevalence of cardiovascular risk factors, and also include nutritional status of the rural population of the area in the study. 

The results of this study showed that daily intake of healthy food groups such as whole grains, fruits, and unsaturated oil, and also unhealthy food groups such as sweets and high fat dairy products, was simultaneously high in the study population. It seems that the food group intake is more related to the tendencies and taste preferences of the study population. Younger people, male subjects and those with lower education use an unhealthier dietary pattern. Therefore, health authorities are recommended to find alternative methods to modify the people's unhealthy nutritional behavior and preferences while they continue to encourage young adults and those with lower education to improve their dietary pattern.
